# Use of insecticide quantification kits to investigate the quality of spraying and decay rate of bendiocarb on different wall surfaces in Kagera region, Tanzania

**DOI:** 10.1186/s13071-015-0859-5

**Published:** 2015-04-22

**Authors:** Narjis G Thawer, Jeremiah M Ngondi, Frances E Mugalura, Isaac Emmanuel, Charles D Mwalimu, Evangelia Morou, John Vontas, Natacha Protopopoff, Mark Rowland, Joshua Mutagahywa, Shabbir Lalji, Fabrizio Molteni, Mahdi M Ramsan, Ritha Willilo, Alexandra Wright, Jessica M Kafuko, Isaiah Ndong, Richard Reithinger, Stephen Masingili Magesa

**Affiliations:** RTI International, Dar es Salaam, Tanzania; Sengerema Health Institute, Sengerema, Tanzania; ACDI/VOCA, Morogoro, Tanzania; National Malaria Control Programme, Dar es Salaam, Tanzania; Liverpool School of Tropical Medicine, Liverpool, UK; Department of Biology, University of Crete, Heraklion, Greece; Faculty of Crop Science, Pesticide Science Lab, Agricultural University of Athens, 11855 Athens, Greece; Institute of Molecular Biology and Biotechnology, Foundation for Research and Technology-Hellas, 73100 Heraklion, Greece; London School of Hygiene and Tropical Medicine, London, UK; Swiss Tropical and Public Health Institute, Dar es Salaam, Tanzania; United States Agency for International Development, Abuja, Nigeria; RTI international, Research Triangle Park, North Carolina, USA; RTI international, Washington, DC USA

**Keywords:** Insecticide quantification kit, Bendiocarb, Indoor residual spraying, Residual life, IRS coverage, Quality of spraying, Tanzania

## Abstract

**Background:**

Bendiocarb was introduced for the first time for Indoor Residual Spraying (IRS) in Tanzania in 2012 as part of the interim national insecticide resistance management plan. This move followed reports of increasingly alarming levels of pyrethroid resistance across the country. This study used the insecticide quantification kit (IQK) to investigate the intra-operational IRS coverage and quality of spraying, and decay rate of bendiocarb on different wall surfaces in Kagera region.

**Methods:**

To assess intra-operational IRS coverage and quality of spraying, 104 houses were randomly selected out of 161,414 sprayed houses. A total of 509 samples (218 in Muleba and 291 in Karagwe) were obtained by scraping the insecticide samples from wall surfaces. To investigate decay rate, 66 houses (36 in Muleba and 30 in Karagwe) were selected and samples were collected monthly for a period of five months. Laboratory testing of insecticide concentration was done using IQK^TM^ [Innovative Vector Control Consortium].

**Results:**

Of the 509 samples, 89.5% met the World Health Organization (WHO) recommended concentration (between 100–400 mg/m^2^) for IRS target dosage. The proportion of samples meeting WHO standards varied between Karagwe (84.3%) and Muleba (96.3%) (p < 0.001). Assessment of quality of spraying at house level revealed that Muleba (84.8%) had a significantly higher proportion of households that met the expected target dosage (100–400 mg/m^2^) compared to Karagwe (68.9%) (p < 0.001). The quality of spraying varied across different wall substrates in both districts. Evaluation of bendiocarb decay showed that the proportion of houses with recommended concentration declined from 96.9%, 93.5% and 76.2% at months one, two, and three post IRS, respectively (p-trend = 0.03). The rate of decay increased in the fourth and fifth month post spraying with only 55.9% and 26.3% houses meeting the WHO recommendations, respectively.

**Conclusion:**

IQK is an important tool for assessing IRS coverage and quality of spraying. The study found adequate coverage of IRS; however, residual life of bendiocarb was observed to be three months. Results suggest that in order to maintain the recommended concentrations with bendiocarb, a second spray cycle should be carried out after three months.

**Electronic supplementary material:**

The online version of this article (doi:10.1186/s13071-015-0859-5) contains supplementary material, which is available to authorized users.

## Background

Malaria remains a huge public health problem in Africa despite substantial measures that have been implemented. The latest report by WHO shows that in 2013, there were 198 million cases of malaria estimated worldwide and 584,000 global malaria deaths of which 90% of the deaths occurred in Africa [1]. Based on a 2011/12 malaria indicator survey, Tanzania has a high burden of malaria with overall prevalence of malaria parasites of 9% (range by region,<1% to 32%) among children under 5 [2]. Efforts to control Malaria have been scaled up in the past few years by the National Malaria Control Programme (NMCP) and its partners which has led to a reduction in Malaria burden [3-7]. Since 2000, insecticide treated nets (ITNs) have been distributed through various campaigns and programmes such as the National Insecticide Treated Nets (NATNETS) Programme [8], the Tanzania National Voucher Scheme introduced in 2004 targeting vulnerable groups such as pregnant women and children aged less than five [9], the children under five catch-up campaign (U5CC) in 2009 [10] and the universal coverage campaign (UCC) in 2011 [11]. In terms of case management, intermittent preventive treatment (IPT-p) was introduced in 2003 and later in 2007, artemisinin-based combination therapy (ACT) was introduced as first-line treatment in all public facilities [5,12]. Also rapid diagnostic tests (RDTs) were introduced in all facilities by 2011 for early detection and treatment of malaria [13]. Moreover, indoor residual spraying (IRS) was introduced in Zanzibar and Kagera regions of Mainland Tanzania in 2006 and 2007 respectively which was later extended to cover the whole Lake Zone in 2011 [14].

IRS is a highly effective intervention for controlling malaria transmission and involves the spraying of insecticides in the interior wall surface of houses to reduce contact between malaria vectors and human hosts [15]. Various insecticide classes such as dichlorodiphenyltrichloroethane (DDT), pyrethroids, organophosphates and carbamates are used for IRS. From 2007 to 2011, blanket IRS was conducted in districts of Lake Zone, which had the highest malaria burden in Tanzania, using pyrethroid lambda-cyhalothrin (ICON®10CS, Syngenta, Basel, Switzerland) [14]. However, following the widespread increase in pyrethroid resistance reported in many African countries [16,17] and in Tanzania [18-20], lambda-cyhalothrin was no longer considered effective. In 2012, an interim plan for insecticide resistance management was developed that recommended change of insecticide class to carbamates; therefore bendiocarb (FICAM® 80 Wettable Powder, Bayer) was introduced for IRS operations [14].

The carbamate was chosen as an alternative due to a number of studies that have shown its use as a substitute for pyrethroid resistance management in IRS operations in Benin [21-24], Equatorial Guinea [25] and Mozambique [26]. However, there were no studies on mosquito vector susceptibility to bendiocarb in Tanzania prior to study by Protopopoff *et.al* (2013) [20]. This insecticide affects the central nervous system by blocking the degradation of the neuromediator acetylcholine through an irreversible acetylcholinestarase inhibitor and is thus lethal against mosquitoes [21,23,27]. Bendiocarb is a carbamate insecticide that is recommended for IRS by the World Health Organization (WHO) since it is safe, odour-free and has the potential to control pyrethroid-resistant mosquitoes [28]. However, the problem with this class of insecticide is its short residual life of 2–3 months requiring at least two rounds of spray cycles per year in order to cover the transmission season(s) [21,29]. An insecticide treatment can only have the desired impact on malaria transmission if houses are properly sprayed and treatment is repeated before insecticide levels fall below their biologically active threshold concentration.

Essential to the success of malaria control campaigns is the implementation of quality control procedures. Due to limited available methods for quantifying the insecticide levels in sprayed houses, the quality of IRS is not frequently assessed. To ensure that IRS is providing the desired protection, it is crucial to verify that surfaces carry sufficient dosage of insecticide and that the required concentration is maintained at the adequate amount before the next round of IRS. The currently available methods for such assessment include high performance liquid chromatography (HPLC) and cone bioassays [30]. Both these methods are expensive, require skilled laboratory staff, are time-consuming and take a long time to generate data, which greatly impinges on the ability of IRS programs to implement quality control procedures. In addition, the HPLC method is subject to bias since the filter papers are placed on the walls prior to spraying operations and are thus visible to the operators [31]. An alternative method, the Insecticide Quantification Kit (IQK), has been shown to be effective for assessing IRS coverage and quality of spraying, and it can effectively monitor insecticide decay rate to establish the right time to conduct a new spray cycle [30]. The feasibility of the kit to estimate cyanopyrethroid levels for IRS in Vanuatu, Tanna Island was evaluated and compared with the HPLC method and the IQK method proved to be more practical for quality control of IRS [30]. The kit has also been demonstrated to be successful in field trials and laboratory testing in Equatorial Guinea, Mozambique and Ethiopia [32]. The kit quantifies the amount of insecticide on a sprayed/treated surface and can therefore support determining the proportion of houses treated within the target dose range, two important components for establishing quality of spray operation.

In this study, the IQK method was used to monitor bendiocarb concentration, investigate the intra-operational IRS quality of spraying, and explore decay rate of the bendiocarb on different wall surfaces.

## Methods

### Study site

This study was conducted in Karagwe (1° 26’ 15” S, 31° 10’ 25” E) and Muleba (1° 50’ 23” S, 31° 31’ 16” E) districts in Kagera region of Northwest Tanzania on the western shore of Lake Victoria. Kagera region is a malaria endemic area and is thus the target for malaria control interventions in Tanzania. From 2007 to 2011, IRS in the region was undertaken using the pyrethroid lambda-cyhalothrin insecticide (ICON®10CS, Syngenta, Basel, Switzerland). In 2012, an interim plan for insecticide resistance management led to change of insecticide to bendiocarb (FICAM® 80WP) for IRS operations [14]. The bendiocarb spray round was performed in January 2012 in Muleba sites and March 2012 in Karagwe sites.

### Selection of houses and sample collection for intra-operational IRS target dosage

From 11 IRS operation sites in Muleba and 9 in Karagwe district, 50% of villages per site were randomly selected followed by selection of 50% hamlets per village. In each selected hamlet, 10% of houses were then selected using systematic random sampling. This resulted in 104 houses being randomly selected out of 161,414 sprayed houses in Karagwe and Muleba districts for quantifying the amount of insecticide applied to the surface of the walls of these houses.

**Figure 1:**
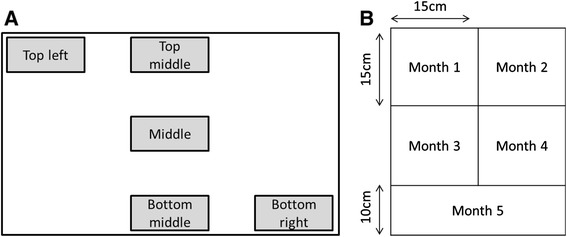
Sample collection for assessing **A)** The quality of spraying and **B)** Decay rate of bendiocarb from the square drawn in the middle of the left wall of the living room.

The amount of insecticide applied to the surfaces was quantified in the 104 randomly selected houses (58 in Karagwe and 46 in Muleba). The samples were collected during the IRS operation after the houses were sprayed with bendiocarb in the first round of spraying for both districts. One sample was collected in the middle of one wall in each room of every house for evaluating IRS coverage. To assess the quality of intra house spraying, five samples were collected in one wall of the living room in each of the randomly selected houses. One on the top left side of the wall, one on the top middle, one on the middle, one on the bottom middle and one on the bottom right (Figure 1A).

### Selection of houses and sample collection for decay rate evaluation

Evaluation of the decay of bendiocarb over time was conducted as an indicator for starting a new spray round whereby periodical follow up of the houses was carried out. Cluster sampling was used for selecting ten houses per substrate wall that included mud wall, burnt bricks, un-burnt bricks, cement plastered, mud-clay wall, lime plastered and sand plastered wall. For the first sample collection i.e. one month after the spraying, additional houses were sampled (20 houses for each substrate). The houses were then tested and only those with the WHO recommended concentration of 100 – 400 mg/m^2^ were selected, while those with low concentration below WHO standards were dropped in the subsequent monthly collection. A total of 66 houses met the WHO standards and were followed up on monthly basis for a period of five months.

One sample per house was collected from the indoor wall surface of the living room using the scratching method. The same wall for every house was selected for scraping to harmonize the collection (left wall). With the head of the household’s consent, a square was drawn with a chalk in the middle of the left wall and divided into 5 sections (Figure 1B). Each of the squares represented the area where the sample was to be collected for the next 5 months post spraying. A questionnaire on the type of material used to construct the houses, IRS and malaria prevention methods used in the house was filled out (see additional file 1). The questionnaire was completed in detail during the collection of the first sample. During the collection of subsequent samples, only information regarding whether or not the wall was re-plastered or washed was collected. Wall substrate samples were obtained by scratching a thin layer of the left wall surface using a scalpel and collecting the powder in eppendorf tubes up to 0.5 ml mark. Each tube was given a unique identification number and this number was recorded in the questionnaire form. The date of collection, site, part of wall and type of substrate was also labeled on the tubes, which were then transported to the analysis site.

### Determination of bendiocarb decay activity, using Insecticide Quantification Kit (IQK)

**Figure 2:**
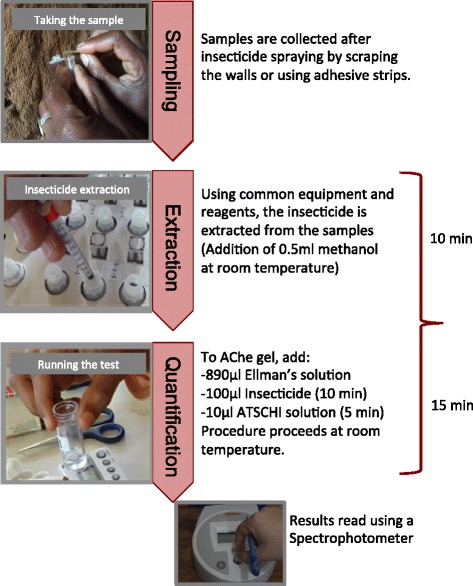
Procedures involved in determining insecticide content after indoor residual spraying using the insecticide quantification kit.

Laboratory testing of insecticide was carried out using the IQK™ [32]. The IQK for bendiocarb quantifies the amount of bendiocarb on a sprayed/treated surface. Figure 2 summarizes the procedures involved for determining insecticide content, that is, sample collection, extraction and quantification. The IQK assay is based on inhibition of the activity of recombinant acetylcholinesterase (AChE) by bendiocarb which is dependent on the concentration of the insecticide [27]. The assay system is composed of a gel-strip that contains the enzyme, AChE immobilized in gel, in cuvettes, and an enzyme substrate solution. The assay protocol involves adding the bendiocarb-containing sample solution to a reaction tube containing the enzyme impregnated gel strip. This is followed by addition of a chromogenic substrate solution. The detectable concentration range of the assay for bendiocarb is 0.1 – > 200 mg/m^2^ (IRS). The assay is sensitive requiring very low (μg) quantities of enzyme.

The kits stored at 4°C were left at room temperature for at least 30 minutes before the start of the test. 0.5 ml of methanol was added to the tubes containing the sample, and mixed. The tubes were then left on the bench for 10 min to ensure maximum insecticide release. 890 μl of Ellman’s solution was added to the cuvette containing the gel impregnated enzyme formulation. This was followed by adding 100 μl of the sample mixed with methanol. For the control test, 100 μl of methanol was added to the cuvette. The cuvettes were then incubated at room temperature for 10 min. In another tube containing 29 mg of Acetylthiocholine iodide powder (ACTCHI), 1 ml of water was added and the solution mixed to dissolve the powder. 10 μl of the ACHTI solution was then added to the cuvette with the gel impregnated enzyme formulation and sample, then incubated for 10 min. The liquid was then removed from the cuvette. The photometer intensity of the yellow colour in the gel impregnated enzyme formulation was read at OD400 and recorded (t = 10 min). Two more measurements were read at t = 20 min and t = 30 min. The OD results at time point 30 min, were classified based on WHO recommendations as shown in Table 1 [31].

**Table 1:** **Scoring criteria based on manufacturer’s recommendations**

**Optical density**	**Concentration range**	**Recommendation**
>0.39	Below 20 mg/m^2^	No residual insecticide
0.3-0.39	20-100 mg/m^2^	Insecticide dose below WHO recommendation
<0.3	100-400 mg/m^2^	Insecticide dose meets WHO recommendation

### Data analysis

Data were entered onto a Microsoft Excel spreadsheet. Statistical analysis was conducted using Stata 12.0 (Stata Corporation, College Station, Texas). Distribution of IRS coverage and intra-house quality was assessed using contingency tables, while differences in proportions were compared using the *χ*^2^ test. Variation in quality of spray was further assessed using box plots and the difference in optical density distribution was tested using Mann–Whitney *U* test.

### Ethical consideration

Ethical approval for this study was granted by the ethics review committees of the Kilimanjaro Christian Medical College, the Tanzanian National Institute for Medical Research, and the London School of Hygiene and Tropical Medicine (Trial registration number: NCT01697852). Written informed consent was obtained from all the household heads where sample collections were carried out.

## Results

### IRS coverage in houses

Table 2 summarizes the results of the samples collected for estimating the insecticide dosage on wall surface study in 104 households. A total of 509 samples were collected, of which four were spoilt and were not analyzed. Of the 505 samples, 89.5% met the WHO recommended concentration for IRS (100 – 400 mg/m^2^) target dosage in both Karagwe and Muleba districts. The proportion of samples that met the WHO standards, varied significantly between Karagwe (84.3%) and Muleba (96.3%) districts (p <0.001).

**Table 2:** **Indoor residual spraying coverage in Karagwe and Muleba districts**

**District**	**Number of households**	Number of samples*(N)	**Proportion of samples (n)**		
			**No residual insecticide**	**Insecticide below WHO recommendation**	**Insecticide meets WHO recommendation**
Karagwe	58	287	12.9% (37)	2.8% (8)	84.3% (242)
Muleba	46	218	1.8% (4)	1.8% (4)	96.3% (210)
Total	104	505	8.1% (41)	2.4% (12)	89.5% (452)

*4 samples spoilt, thus not analysed.

### Quality of spraying

Table 3 shows the variation of insecticide concentration levels within the same house by district. In Karagwe district, 68.9% of houses had walls that completely met the WHO requirements of insecticide levels indicating good quality of spraying. In the remaining proportion of houses, 25.9% had walls with varying levels of insecticide while 5.2% of houses had sections on the wall that had no insecticide at all. No such houses were recorded in Muleba. In Muleba, 84.8% of houses had good quality of spraying meeting the WHO standards whilst the remaining 15.2% of houses had walls with varying levels of insecticide. The proportion of households that met the WHO requirements of insecticide levels was significantly higher in Muleba compared to Karagwe district (p <0.001).

**Table 3:** **Variation in quality of spraying in Karagwe and Muleba districts**

**District**	**Number of households**	**Proportion of households (n)**		
		**No residual insecticide**	**Varying levels of insecticide**	**Insecticide meets WHO recommendation**
Karagwe	58	5.2% (3)	25.9% (15)	68.9% (40)
Muleba	46	0.0% (0)	15.2% (7)	84.8% (39)
Total	104	2.9% (3)	21.2% (22)	75.9% (79)

**Figure 3:**
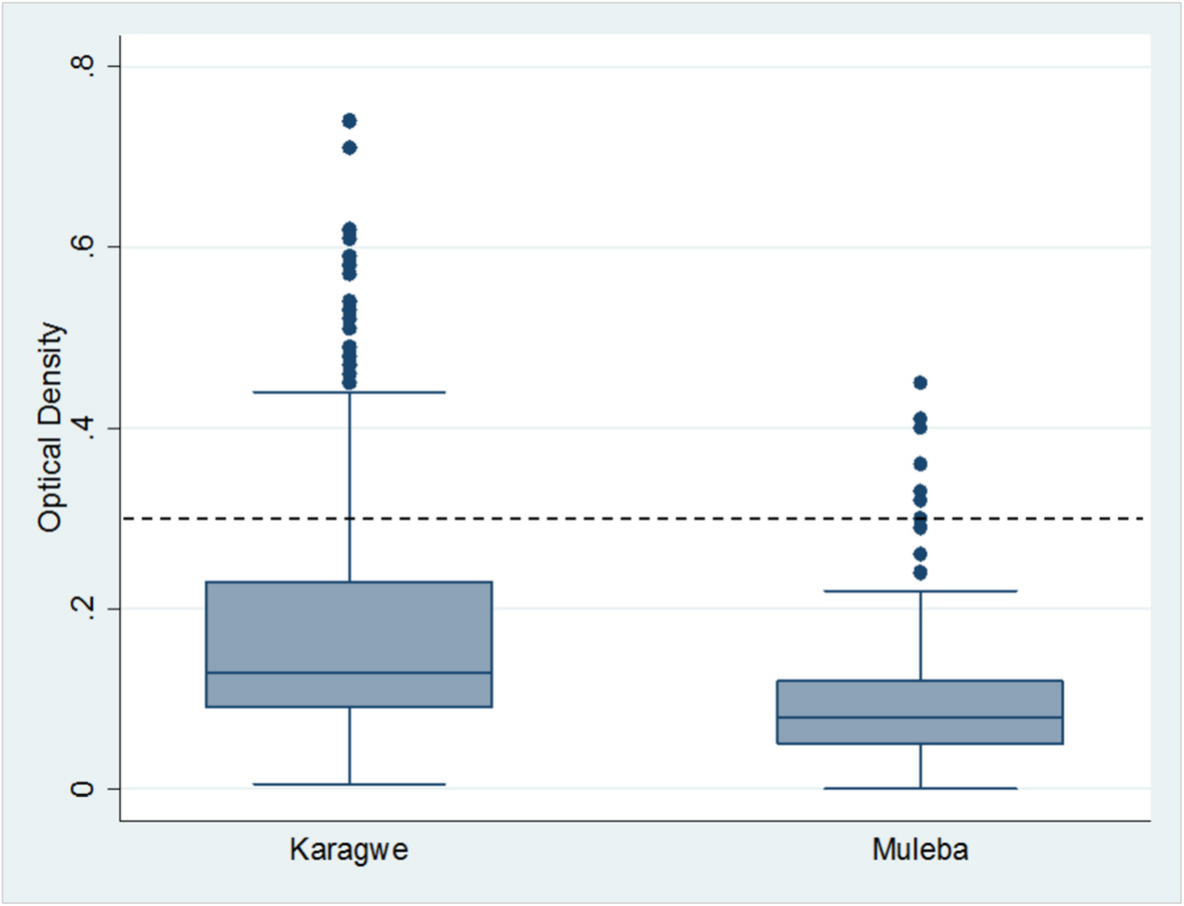
Box plots showing the distribution of the concentration of bendiocarb in Karagwe and Muleba districts to determine the variation in the quality of spraying. The dashed line represents the recommended WHO threshold level of less than 0.3.

Figure 3 shows the distribution of insecticide concentration in the houses by district. In Muleba, most of the houses met the WHO recommended optical density (OD) of less than 0.3 and there was a statistically significant difference in the distribution of OD between Muleba and Karagwe (Mann–Whitney *U* test, p <0.001).

**Figure 4:**
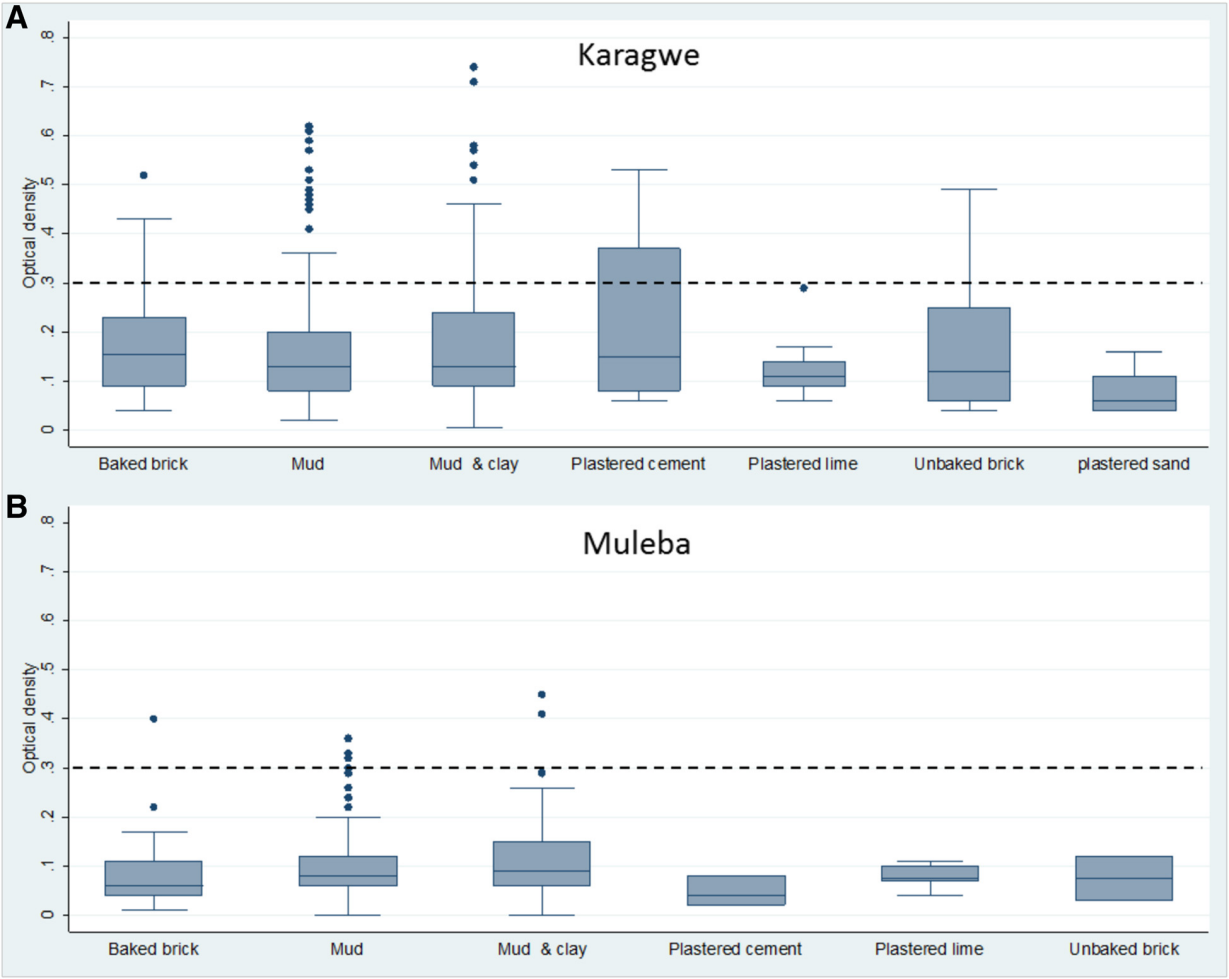
Box plots showing the distribution of the concentration of bendiocarb in different wall substrates in Karagwe **(A)** and Muleba **(B)** districts. The dashed line represents the recommended WHO threshold level of less than 0.3.

Figure 4 shows the concentration of the insecticide for different wall substrates in each district. The quality of spraying varied across the different wall types in Karagwe (Figure 4A) and Muleba (Figure 4B). In Muleba, most wall types had good quality of spraying that met the WHO recommendations. In Karagwe, all wall surface types had a substantial number of households below the WHO recommended levels (100 – 400 mg/m^2^), except for sand plaster and lime plaster surfaces.

### Bendiocarb decay

**Figure 5:**
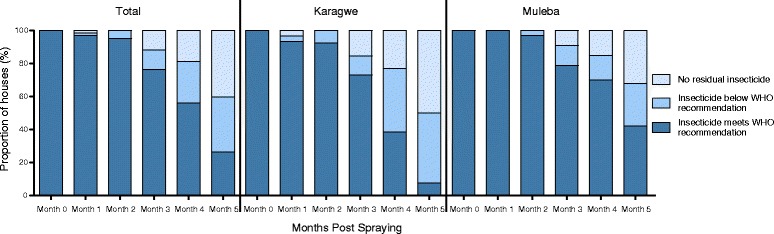
Decay rate of bendiocarb over five months post spraying showing the proportion of houses with different insecticide levels at each month displayed for both districts combined (Total) and individual (Karagwe and Muleba) districts.

To determine the decay rate of bendiocarb, 66 houses (36 in Karagwe and 30 in Muleba) that had the recommended insecticide concentration at baseline (Month 0) were followed up over a period of 5 months. The results showed a slow rate of decay in Month 1 and Month 2 but a rapid decay from Month 3 onwards. Overall, the proportion of houses that met the WHO standards declined to 96.9%, 93.5% and 76.2% at months one, two, and three post IRS, respectively (p-trend = 0.03). The rate of decay increased in the fourth and fifth months post spraying with only 55.9% and 26.3% houses meeting the WHO recommendations, respectively (Figure 5). Karagwe had a significantly higher decay rate compared to Muleba in Month 4 (38.5% vs. 69.7% that met the WHO standards; p-value = 0.047) and Month 5 post spraying (7.7% vs. 41.9% that met the WHO standards; p-value = 0.014) (Figure 5).

## Discussion

Our study assessed the amount of bendiocarb deposited on different wall surfaces during IRS in Karagwe and Muleba districts of Kagera region, Tanzania. In addition, the insecticide decay rate was monitored to establish the right time to conduct a new spray cycle. High coverage of IRS provides the best protection from malaria infection. Only when 80% of coverage with target dosage is achieved, can the IRS be considered effective [31]. The results of this study showed that the proportion of houses that met WHO standards for IRS coverage was 89.5% indicating high coverage. Adequate coverage varied by location with Muleba (96.3%) having a significantly higher IRS coverage as compared to Karagwe (84.3%). Heterogeneity in bendiocarb content was observed in both districts with the distribution of the variation in spray dosage being higher in Karagwe. Parts of the wall within some houses had insecticide levels that met the expected target dosage (100–400 mg/m^2^) while other sections of the same wall had levels below the target dosage or with no residual insecticide at all. Hence, this variation in quality of spraying may explain the reduced IRS coverage observed in Karagwe. Different factors may have contributed to the variation in the insecticide levels such as the method of spraying not being consistent by the spray operators, poor spraying techniques and sprayer not observing pump pressure or use of worn out nozzles. Measures to improve the quality of spraying need to be implemented to ensure that the insecticide content does not reach a level whereby it becomes ineffective and offers little or no protection at all.

This study also assessed the quality of bendiocarb spraying across different wall substrates. It was found that bendiocarb levels varied across different wall types with walls made of plastered lime showing the best quality of spraying in both the districts. In Muleba, most wall types had good quality of spraying that met the WHO recommendations. In Karagwe, five of the wall types had a substantial number of households with insecticide deposit below the WHO recommended levels, except for plastered sand and plastered lime surface types. These results suggest that the difference in the quality of spraying between districts was not a function of the type of wall substrates found in the district.

It is important to identify the residual life of the insecticide used in order to plan an effective IRS operation and establish the right time for re-spraying. In this study, the decay rate of bendiocarb showed a slow rate of decay in the first 3 months with the majority of houses meeting the WHO recommended target insecticide dosage. However, the rate of decay increased in the fourth and fifth month post spraying. This is consistent with efficacy studies conducted in south Cameroon [33], Benin [21], Bioko Island and Equatorial Guinea [29], which showed that the residual life of bendiocarb was less than four months. It is thus necessary to have at least 2 to 3 treatment rounds per year to ensure that sufficient dosage of the insecticide is present on the walls and this is crucial to achieve a long term efficacy for bendiocarb in IRS. However, this would add to the cost of such an intervention considerably as bendiocarb is an expensive insecticide and the operational costs would also double or even triple depending on the number of rounds conducted [34]. Developing a microencapsulated formulation of the bendiocarb may make it have a longer residual life [35]. Moreover, it is important to note that just like resistance to pyrethroids occurred, repeated use of carbamates in the same area could lead to resistance to carbamates, which has been reported before [20,36,37]. Hence, close monitoring of carbamate insecticides will be required to detect the presence of vector resistance so that appropriate resistance management strategies can be implemented.

Due to the limited availability of methods for quantifying the insecticide levels in sprayed houses, the quality of IRS is not routinely assessed. This study used the IQK method to assess the amount of insecticide applied on wall surface, IRS coverage and quality of spraying. However, one of the potential limitations of the IQK method was to develop an accurate method to extract sample insecticide from the walls of the sprayed houses taking into account the different wall substrates. The use of adhesive tapes as recommended by the manufacturer to collect samples picked extremely low quantities of wall surface substrate material as reported before [30]. Thus, scratching of wall surfaces proved effective for sampling the insecticide. The other limitation was the presence of outliers in the data set that could affect the accuracy of the results. These outliers could have been due to sampling errors or technical kit errors, which may be reduced by being more consistent in sampling and quantification procedures, collecting more samples from different sites and taking replicates of the same sample for better accuracy.

The IQK assay proved to be a useful tool as it was a quick method for assessing IRS quality and coverage of spraying and for following up the carbamate decay rate with time. The IQK was able to detect presence of carbamate on wall surfaces within the prescribed time (<30 min/sample). The kit could show the various insecticide concentrations scored as optical density and provided a rapid assessment of the performance of the IRS operation. The results were immediately available and interpreted by the staff in the field, to evaluate the spray coverage, quality of the operation and monitor the rate of bendiocarb decay. A similar IQK method that incorporated a rapid colorimetric assay was used for estimating cyano-pyrethroid levels for IRS in Vanuatu [30]. The kit proved to be simple to perform and was recommended for routine quality control in malaria control programs.

## Conclusions

IQK is an important tool for assessing IRS coverage and quality of spraying. Bendiocarb insecticide was found to have high IRS coverage that is necessary to significantly reduce human-vector contact. The concentration of insecticide varied with different wall substrates and it is thus important to monitor the quality of spraying to ensure that appropriate concentrations of insecticide are sprayed on the walls. However, the residual life of bendiocarb was observed to be three months and therefore, at least two rounds of spray cycles per year are required for the insecticide to be effective.

## Additional file

Additional file 1:
**Questionnaire for IQK study.**

## Abbreviations

IRS: Indoor Residual Spraying
IQK: Insecticide quantification kit
WHO: World Health Organization
NMCP: National Malaria Control Programme
LLIN: Long-lasting insecticidal nets
DDT: Dichlorodiphenyltrichloroethane
HPLC: High performance liquid chromatography
AChE: Acetylcholinesterase
ACTCHI: Acetylthiocholine iodide
OD: Optical density
