# Supplementary material for: Use of insecticide quantification kits to investigate the quality of spraying and decay rate of bendiocarb on different wall surfaces in Kagera region, Tanzania
Source: Parasit Vectors. 2015 Apr 22;8:242. doi: 10.1186/s13071-015-0859-5 (PMC4424830; doi:10.1186/s13071-015-0859-5)
Supplement: Additional file 1: — Questionnaire for IQK study. [file 13071_2015_859_MOESM1_ESM.docx]

**Questionnaire for IQK study**
